# Validation of an IFN-gamma ELISpot assay to measure cellular immune responses against viral antigens in non-human primates

**DOI:** 10.1038/s41434-020-00214-w

**Published:** 2021-01-11

**Authors:** Fan Yang, Kathryn Patton, Theresa Kasprzyk, Brian Long, Soumi Gupta, Stephen J. Zoog, Kristin Tracy, Christian Vettermann

**Affiliations:** BioMarin Pharmaceutical, Inc, Novato, CA USA

**Keywords:** Gene therapy, Biomarkers

## Abstract

Adeno-Associated Virus (AAV)-based gene therapy vectors are in development for many inherited human disorders. In nonclinical studies, cellular immune responses mediated by cytotoxic T cells may target vector-transduced cells, which could impact safety and efficacy. Here, we describe the bioanalytical validation of an interferon-gamma (IFN-γ)-based Enzyme-Linked Immunospot (ELISpot) assay for measuring T cell responses against viral antigens in cynomolgus monkeys. Since ELISpots performed with antigen-derived peptides offer a universal assay format, method performance characteristics were validated using widely available peripheral blood mononuclear cells (PBMCs) responsive to cytomegalovirus peptides. The limit of detection and confirmatory cut point were established using statistical methods; precision, specificity, and linearity were confirmed. Monkey PBMCs from an AAV5 gene therapy study were then analyzed, using peptide pools spanning the vector capsid and transgene product. AAV5-specific T cell responses were detected only in 2 of 18 monkeys at Day 28, but not at Day 13 and 56 after vector administration, with no correlation to liver enzyme elevations or transgene expression levels. No transgene product-specific T cell responses occurred. In conclusion, while viral peptide-specific IFN-γ ELISpots can be successfully validated for monkey PBMCs, monitoring peripheral T cell responses in non-clinical AAV5 gene therapy studies was of limited value to interpret safety or efficacy.

## Introduction

Increasing numbers of gene therapies are currently being developed and hold the potential for a long-term amelioration for many genetic diseases, such as hemophilia [1–4], phenylketonuria [5], lipoprotein lipase deficiency [6], and muscular dystrophy [7, 8]. Adenovirus-associated virus (AAV)-based vectors are commonly used for gene delivery to various target tissues and are generally well tolerated [9]. However, pre-existing as well as treatment-induced immune responses against both AAV capsids and encoded transgene products have been reported in the literature, which may negatively impact safety and efficacy of gene therapies [10–12]. Therefore, treatment-specific humoral and cellular immunity are closely monitored in both clinical and nonclinical studies in order to identify potential barriers to successful gene therapy outcomes [13–15].

Functional measures of antigen-specific cellular immune responses can be achieved by detection of T cell activation, such as cytokine production, cell proliferation, or cytotoxicity. Detection of cytokine secretion as a direct marker for T cell recognition of specific antigens is often preferable for ease of use [16, 17]. Enzyme-Linked Immunoassay and Enzyme-linked Immunospot (ELISpot) assays can both detect secreted cytokines, but they differ by either measuring bulk cytokine production or enumerating individual cytokine-producing cells, respectively. Interferon-gamma (IFN-γ) is often used as a cytokine endpoint in ELISpot assays and has a wide range of biological functions in the context of an immune response, including the activation of macrophages, up-regulation of major histocompatibility complex I and II, and induction of IgG production from activated B cells. IFN-γ is mainly produced and secreted by activated CD4 and CD8 T cells, which constitute about 45–70% of PBMCs in human peripheral blood [18–21]. PBMCs are easily isolated from blood using standardized procedures and can be obtained at any time during a study.

Since the ELISpot technique was introduced, it quickly became a widely applied method for the detection of cellular immune responses [22, 23], for examples in immunodeficiency diseases and vaccine development [24–28]. The method has a relatively wide quantitative range and offers unique sensitivity by revealing cytokine secretion at the single-cell level. In recent years, its application has been adopted by the gene therapy field [29]. A number of clinical studies have used IFN-γ ELISpot assays to evaluate preexisting T cell immunity against multiple AAV serotypes [30, 31]. Other studies monitor for the development of cellular responses to the AAV capsid and transgene encoded protein following dose administration [1, 13, 32]. IFN-γ ELISpot assays have also been used for preclinical evaluation of immunity to AAV vectors [33, 34]. However, there are limited studies to help standardize the bioanalytical assessment of method performance characteristics that are required for regulated clinical or nonclinical sample testing [19, 35, 36]. For instance, it is a matter of choice how to evaluate ELISpot detection cutoffs (limit of detection, LOD), whether to implement confirmatory cut points, or how to set assay acceptance criteria for precision during validation and sample analysis. In contrast, these types of questions have been addressed and standardized for traditional immunogenicity assays that detect total or neutralizing antibodies (TAbs or NAbs) as well as for quantitative ligand-binding assays used for assessment of biomarkers and pharmacokinetics [37–42]. Given their cell-based format, ELISpot assays most closely resemble assays used to detect NAbs, and we expect future regulatory guidance for ELISpots to reflect this fact.

Here, we describe the development and formal bioanalytical validation of an IFN-γ ELISpot assay, using CMV-derived peptide pools to evaluate various bioanalytical method parameters. Industry white papers [43–46] and some of the above mentioned guidance documents for regulated bioanalytical assays were considered wherever possible. The method was based on a commercial ELISpot assay kit for monkey IFN-γ detection, as cynomolgus monkeys are commonly used in non-clinical gene therapy studies to assess safety and efficacy. During assay establishment, basic assay parameters were optimized for image capture, cell density, detection antibody concentration, and stimuli concentration to achieve optimal performance for monkey PBMC samples (data not shown). Initial assay characterization included intra- and inter-assay precision and verification of a preliminary LOD. Formal assay validation confirmed intra-assay, inter-assay, and inter-analyst precision, linearity and specificity, and statistically derived an LOD and confirmatory cut point. The validated assay was considered fit-for-purpose and used in a monkey study to measure cellular immune responses to the AAV5 vector capsid and encoded transgene product following gene therapy administration.

## Materials and methods

### Cynomolgus monkey PBMC preparation

Naïve Cynomolgus monkeys (*Macaca fascicularis*) whole blood was purchased from Valley Biosystems (West Sacramento, CA) and HumanCells Biosciences (Sunnyvale, CA). Monkey PBMCs were isolated from sodium heparin anticoagulated whole blood as previously described with some modifications [47]. Any potential stimulation factors were absent during PBMC isolation and storage, for example by avoiding exposure to FBS or other growth factors. This requirement was critical to avoid non-specific stimulation. Briefly, PBMCs were isolated from whole blood within 6 h after collection. Blood volume was diluted 1:1 with 1X Ca^2+^/Mg^2+^ Free DPBS (14190–444, Gibco). For instance, 5 mL of blood should be gently mixed with 5 mL of 1X Ca^2+^/Mg^2+^ Free DPBS. This mixture was layered onto room temperature Lympholyte®-Mammal (CL5120, Cedarlane) or other similar reagent at a 75% Lympholyte to DPBS-diluted blood volume ratio. To illustrate, 10 mL of diluted blood was layered onto 7.5 mL Lympholyte®-Mammal. Samples were centrifuged at 1270–1450 RCF for 30 min at room temperature with the brake off. The PBMCs were harvested after centrifugation, washed, diluted in either CryoABC (CTLC-ABC, Cellular Technology Limited) or CryoStor CS10 (07930, StemCell Technologies) serum-free freezing medium and aliquoted for freezing. PBMC aliquots were frozen slowly at −80 °C in Mr. Frosty cell freezing containers (Thermo Scientific). The samples were transferred into LN_2_ (vapor phase) within 24–96 h and remained there until shipment. Frozen vials of PBMCs were stored in an LN_2_ tank until the assay started.

### Antigen and control preparation

Monkey cytomegalovirus (CMV) peptide pools UL55 (PM-CyCMV-UL55), UL83 (PM-CyCMV-UL83), and human CMV peptide pools pp65 (PM-PP65–1) were purchased from JPT Peptide Technologies (Berlin, Germany). Human CEF (CMV, Epstein-Barr virus, Influenza virus) peptide pool (3615–1) was purchased from MabTech (Cincinnati, OH, USA). AAV5 peptide pools (15mers overlapping by either 10 or 11aa) with ≥80% purity were custom synthesized and ordered from JPT. Each AAV5 peptide pool (AAV5pp1 and AAV5pp2) consisted of ~70–90 overlapping 15-mer peptides that were synthesized based on the sequence of AAV5 viral capsid protein 1 (VP1). AAV5 viral capsid protein 2 (VP2) and AAV5 viral capsid protein 3 (VP3) are also covered by these peptides pools since they are shorter version of VP1 with otherwise identical peptide sequences. Human immunodeficiency virus envelope (HIV) peptide pool (PM-HIV-ENV, JPT Peptide Technologies) was used as a non-responsive peptide pool control. For each peptide pool, the lyophilized vial was reconstituted with DMSO and CTL-Test media (Cellular Technology Limited (CTL), catalog #CTLT-005) to achieve a stock concentration at 8–200 µg/mL (contains 1% DMSO) and stored in single-use aliquots at −20 °C. The final concentration of DMSO in the assay was between 0.1 and 0.25% (final in-well concentration). Phytohemagglutinin-L (PHA-L), a mitogen and a lectin from the red kidney bean (*Phaseolus vulgaris*), was used as a positive control stimulant. A vial of PHA-L (L2769, Sigma Aldrich) was reconstituted to achieve a 1 mg/mL stock solution by adding sterile 1X PBS, aliquoted and stored at −80 °C. The mock stimulation, or negative control, contained an equivalent concentration of DMSO as contained in the peptide pools (1X concentration: 0.1–0.25% DMSO).

### IFN-γ ELISpot assay procedure

IFN-γ ELISpot assays were performed using the Monkey IFN-γ ELISpot PLUS ALP kit (3421M-4APW, Mabtech) according to the manufacturer’s protocol with some modifications. Basic assay pipetting workflow was performed as follows; the assay plate was re-hydrated by washing with 1X PBS and blocked with CTL-Test media (CTLT-005, Cellular Technology Limited) for 2–6 h at RT. Meanwhile the stimuli were prepared at 2X and, to each well of a blocked ELISpot plate, 100 µl of 2X stimuli was added in triplicate for each PBMC sample to be tested. Briefly, the 2X concentrations of all stimuli were prepared as follows: peptide pool stimuli were diluted to either 4 µg/mL (0.5% DMSO) or 2 µg/mL (0.25% DMSO), 0.25 ug/mL for the PHA-L positive control, 0.2–0.5% DMSO for the unstimulated negative control, and CTL-Test media alone for the background negative control (containing medium only without PBMCs). Freshly thawed PBMCs from one or more frozen vials were thawed quickly at 37 °C and washed in CTL-Wash media (CTLW-010, Cellular Technology Limited) supplemented with 50 U/mL Pierce Universal Nuclease (88701, ThermoFisher). The PBMC were counted using trypan blue on the ViCell XR (Beckman Coulter) or using acridine orange/propidium iodide stain (CS2–0105, Nexcelom) on the Cellometer (Nexcelom). PBMC, with a viability ≥70%, were diluted in CTL-Test media to a concentration of 2.0 × 10^6^ PBMC/mL and plated 100 µl/well in triplicate for each stimulus (2.0 × 10^5^ PBMC/well). PBMCs were stimulated for 18–24 h at 37 °C + 5% CO_2_ after which cells were removed and plates were incubated with a streptavidin alkaline phosphatase-coupled detection antibody for 1 h. Spots were visualized after incubation with the substrate BCIP/NBT-plus for 3 min ± 15 s at RT. Plate development was stopped with a water wash and the plate was air-dried overnight at RT, avoiding exposure to light. Spots were enumerated using an automated spot counter (ImmunoSpot® CTL S6 Micro Analyzer; Cellular Technology Limited) within 24–96 h and spot counts are further analyzed as spot forming units (SFU) per well. The Immunospot settings used were a sensitivity of 145 or 187, background balance of 10, spot separation of 1–3, and spot size minimum of 0.0015 to maximum of 9.6296 or 9.6466 mm^2^. These parameters were optimized for resolution and accurate enumeration of spots. Raw data included images for each well for visual quality checking and the corresponding spot forming unit (SFU) counts per well.

### Statistical analysis

The percent coefficient of variation (CV) was calculated from the estimated standard deviation and the mean SFU/well responses for assay precision. During LOD determination, the Shapiro–Wilk test was used to test for normality of the data distribution for mean DMSO responses and cut point ratio data collected from 18 donors. The Prob < W value listed in the output is the *p* value. If the chosen alpha level is 0.05 and the *p* value is <0.05, then the null hypothesis that the data are normally distributed is rejected. If the *p* value is >0.05, then the null hypothesis is not rejected [48]. The interquartile-range (IQR) rule was applied to identify statistical outliers: IQR = Q3 – Q1 was multiplied by 1.5 and either added to Q3 or subtracted from Q1; any number above or below these limits was considered a statistical outlier.

### Cynomolgus monkey study design and ethical standards

Cynomolgus monkeys from Charles River Laboratories (Reno, NV) were used for safety evaluation of an AAV5-based gene therapy. Subjects were randomized before being assigned to AAV5 treatment (investigational drug) or control (drug vehicle - buffer) groups. Blood samples were collected by venipuncture at different time points as scheduled (pre-dose sample at day 13, after dose at day 28 or day 56, depending on the group assignment), where PBMCs were isolated within 6 h to ensure optimal T cell function and activity. Subsequently, PBMCs were stored in liquid nitrogen until the ELISpot assay was performed. All procedures were conducted in accordance with all applicable sections of the Final Rules of the Animal Welfare Act regulations (Code of Federal Regulations, Title 9), the Public Health Service Policy on Humane Care and Use of Laboratory Animals from the Office of Laboratory Animal Welfare, and the Guide for the Care and Use of Laboratory Animals from the National Research Council. The protocol and any amendments or procedures involving the care or use of animals in this study were reviewed and approved by the Testing Facility Institutional Animal Care and Use Committee before the initiation of such procedures.

## Results

### Assay establishment and preliminary characterization of LOD

**Fig. 1:**
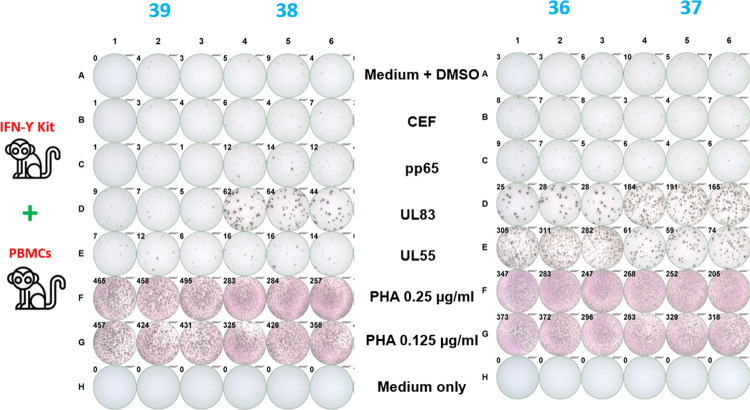
Examples of scanned images and SFU/well counts for stimulated and unstimulated monkey PBMCs. Medium only without PBMCs yielded zero counts. Medium with DMSO (mock control) consistently demonstrated ≤10 SFU/well across all monkeys. Positive responses were observed for PHA stimulation across all monkeys, which served as a pan-T cell stimulation control for the functionality of PBMCs. Three monkeys (36, 37, 38) responded positively when stimulated with monkey CMV peptide pools (UL55 and/or UL83); no or low responses were observed in any monkey when stimulated with a human CMV peptide pool (pp65) or other human virus-derived peptide pools (CEF).

Cytomegaloviruses (CMVs) are endemic in cynomolgus monkey populations. Thus, CMV-derived peptides are ideal antigens for positive control stimulation when testing monkey PBMC samples. To confirm this assumption, PBMCs from five monkeys were stimulated using two different peptide pools, derived from either tegument phosphoprotein UL83 or glycoprotein UL55 of cynomolgus macaque-specific CMV. PBMCs isolated from three monkeys responded to monkey CMV-derived peptide pools (UL83 and UL55), while only low to no responses were observed to a corresponding human CMV-derived peptide pool (pp65) in any monkey sample. Representative images and spot counts are shown in Fig. 1.

**Fig. 2:**
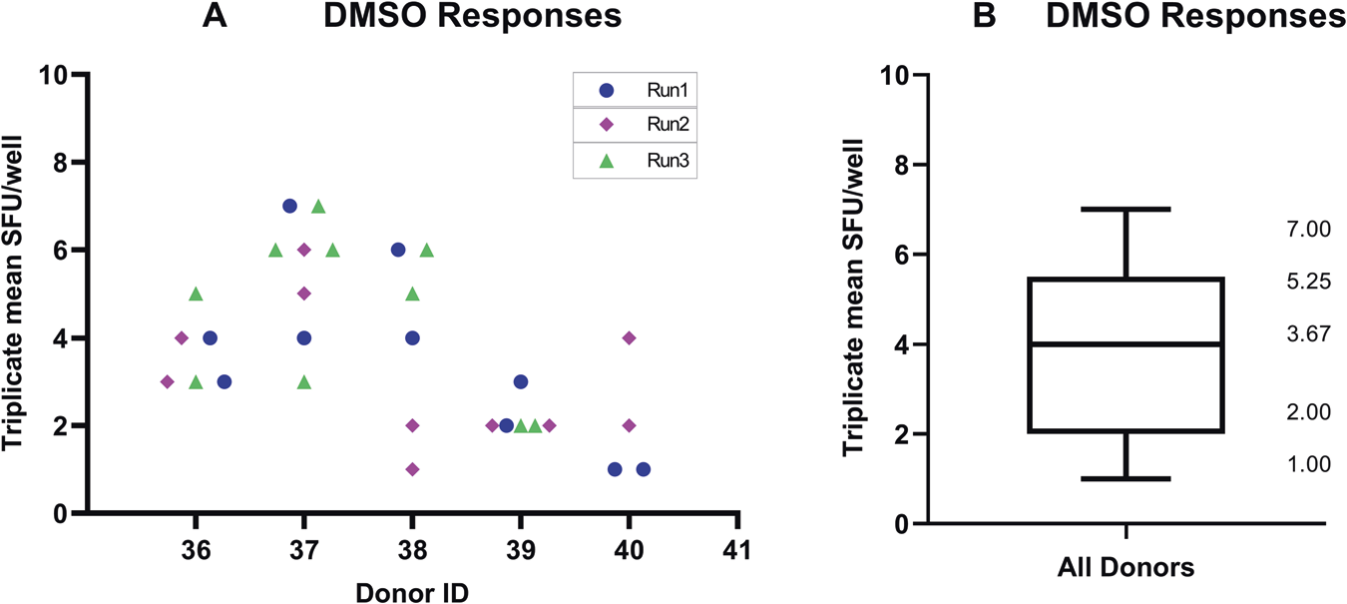
LOD characterization using responses in unstimulated PBMCs. **A** A total of 30 triplicate means for mock responses in unstimulated PBMCs were determined for five different monkeys. **B** Triplicate mean responses obtained in unstimulated PBMCs ranged from 1 to 7 SFU/well, with an overall mean response of 3.67 SFU/well (rounded to 4 SFU/well). The upper 75% and lower 25% quartiles of the distribution of triplicate mean responses in unstimulated PBMCs are also shown.

To determine a preliminary LOD, the magnitude of the observed non-specific background signal was assessed using PBMCs from five monkeys under unstimulated conditions (DMSO mock control). Each animal was tested in two sets of triplicate wells per plate in three independent runs (only two independent runs performed for animal 40, 4 sets of triplicates performed for animal 37 in run 3) over five different days. The mean mock response across triplicate wells ranged from 1 to 7 SFU/well, with an average of 4 SFU/well (Fig. 2A, B). In addition, mock responses in individual wells remained ≤10 SFU/well (only one well showed 10 SFU/well). Based on these non-specific background signals, 10 SFU/well was set as the preliminary LOD in the assay and applied to subsequent experiments. Cell viability for all PBMC lots was >70% and spot counts for PHA stimulation were larger than the LOD in all runs, which satisfied assay acceptance criteria.

### Preliminary characterization of intra- and inter-assay precision

**Fig. 3:**
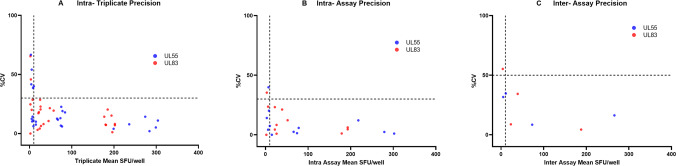
Initial characterization of assay precision. PBMCs from three positive and one negative responders were stimulated with two CMV peptide pools (UL55 and UL83) in three different runs (two sets of triplicates per run) and responses were measured as SFU/well. **A** Intra-triplicate %CVs were plotted against intra-triplicate mean SFU/well responses following UL55 (blue dots) and UL83 (red dots) peptide pool stimulation. **B** Intra assay %CVs were plotted against intra-assay mean SFU/well responses following UL55 (blue dots) and UL83 (red dots) peptide pool stimulation. **C** Inter-assay %CVs were plotted against the inter-assay mean SFU/well responses following UL55 (blue dots) and UL83 (red dots) peptide pool stimulation. In all panels, the horizontal dotted lines represent the pre-defined acceptable %CV limit, and the vertical dotted line represents the preliminary LOD value (10 SFU/well).

Precision was initially characterized using CMV-responsive PBMCs from three different monkeys in three independent runs over 4 days by one analyst, using stimulation with UL83 or UL55 peptide pools at a final concentration of 2 μg/mL. Cell viability for all PBMC lots was >70% and spot counts for PHA stimulation were ≥ LOD in all precision runs (data not shown). For both UL83 and UL55 stimulation, assay precision increased with increasing spot counts, as shown by a trend toward lower CV values when plotted against the mean SFU/well response (Fig. 3A–C). When gated on responses ≥ LOD, the highest intra-triplicate CV was 40.0% (Fig. 3A), the highest intra-assay CV was 23.2% (Fig. 3B), and the highest inter-assay CV was 34.7% (Fig. 3C). Of note, the intra-triplicate CV remained below 30%, when gated on responses ≥30 SFU/well (Fig. 3A). Overall, these results demonstrate that stimulation with monkey CMV-derived UL83 and UL55 peptide pools yielded precisely quantifiable spot counts across a wide range of low to high antigen-specific responses.

### Validation of LOD using statistical methods

After the initial characterization, the ELISpot assay was formally validated using pre-specified acceptance criteria (Table 5). To establish a validated LOD, the magnitude of non-specific background signals observed without any peptide stimulation was evaluated across individual monkeys. This was assessed similarly to how this parameter had been assessed during initial assay characterization but using a larger number of animals and statistical analysis. To this end, PBMCs (viability ≥ 70%) from 18 animals were plated at 200,000 cells/well in triplicate wells in medium containing 0.25% DMSO (mock control) and tested once in a total of four assay runs performed by two different analysts. The triplicate mean responses in SFU/well for the DMSO mock responses were determined for all animals and used to statistically establish the LOD. One data point (71 SFU/well for Subject 7) was identified as a statistical outlier using the interquartile range method and thus excluded from the LOD analysis. The Shapiro–Wilk test using data from the remaining 17 animals showed that values were not normally distributed; therefore, the values were log_10_-transformed, resulting in a normal distribution (Table 1). The LOD was then derived as the 95th percentile upper distribution limit using the formula: anti-log [grand mean + (1.645 × SD) of log-transformed values] and rounded to the nearest whole number. The validated LOD was calculated as 15 SFU/well, which is equivalent to 75 SFU/million PBMCs.

**Table 1:** Validation of LOD and confirmatory cut point.

LOD determination—Mean and ratio					
Mean DMSO responses			Confirmatory response ratios		
Mean SFU/well			Response ratios: antigen vs DMSO		
Donor ID (*n* = 18)	Triplicate mean SFU/well	Triplicate mean SFU (log _10_ transformed)	AAV5 pool 1/mock	AAV5 pool 2/mock	Transgene-product peptide pool/mock
1	2	0.368	1.000	0.714	0.857
2^C^	1	0.000	3.000	1.333	1.667
3^D^	1	0.000	0.000	1.333	2.333
4	1	0.125	1.000	0.250	1.000
5	14	1.146	1.643	1.500	0.952
6	10	1.000	1.600	1.133	1.367
7^A^	71	1.851	1.164	1.192	1.169
8	5	0.699	1.067	1.067	1.000
9	3	0.523	1.700	1.700	1.000
10^B^	1	0.125	2.500	3.750	4.750
11	5	0.727	0.813	1.000	1.125
12	2	0.222	1.000	1.400	1.400
13	9	0.938	1.038	0.577	1.731
14	11	1.041	1.091	0.970	0.727
15	4	0.602	0.917	1.083	1.417
16	8	0.885	1.000	0.826	1.000
17	2	0.368	0.857	0.286	1.143
18	5	0.669	0.286	0.643	0.643
Minimum		0			0
25% percentile		0.17			0.857
Median		0.60			1.000
75% percentile		0.91			1.333
Maximum		1.15			1.731
Mean		0.56			1.049
Std. deviation		0.38			0.387
LOD determination (normal distrib.)			Confirmatory cut point determination		
Mean + 1.645x StDev	1.175		Mean +1.645x StDev		
LOD (Anti-log of above)	**15.0**				**1.69**

Bold numbers represent the final calculated value for LOD and confirmatory cut point ratio respectively.

LOD and confirmatory cut point were based on a statistical analysis of the mean responses (SFU/well) under unstimulated (mock) conditions and the antigen/mock response ratios in naive monkeys, respectively. One result (7A (71)) was identified as a statistical outlier and excluded from the LOD analysis. Response ratios were obtained by dividing the triplicate mean SFU/well responses for AAV5 or transgene-product (TGP) peptide pools by the respective triplicate mean SFU/well response under unstimulated conditions (i.e., peptide stimulation/mock control). Five response ratios (2C (3.000), 3D (2.333) and 10B (2.500, 3.750 and 4.750)) were identified as statistical outliers and excluded from the confirmatory cut point analysis.

### Validation of confirmatory cut point

In 4 of the 18 individual monkeys from the above section, the triplicate mean SFU/well result for unstimulated PBMCs (mock control) fell at or above the LOD (Table 1). Therefore, a confirmatory cut point was required to ensure the antigen-specificity of any potential peptide responses observed for PBMC samples with higher than usual non-specific background. To this end, PBMCs from naïve monkeys were stimulated with two AAV5-derived peptide pools or a transgene product (TGP)-derived peptide pool, each at 2 µg/mL. Response ratios were calculated by dividing the SFU/well results for peptide-stimulated conditions by the SFU/well result for the respective mock control.

To determine the confirmatory cut point, the response ratios from all three peptide stimulations were pooled across all subjects (*n* = 54 data points). Five data points (3.000, 2.333, 2.500, 3.750, and 4.750) from a total of three subjects (subjects 2, 3, and 10) were identified as statistical outliers by the interquartile range method and removed from the confirmatory cut point analysis. Using the remaining 49 data points, the Shapiro–Wilk test showed that the data came from a normal distribution. Therefore, the confirmatory cut point was determined as the 95th percentile distribution limit by determining the grand mean + (1.645 × SD). The confirmatory cut point derived from the response ratios was calculated as 1.69 (Table 1) and will be used to adjudicate sample responses to peptides ≥LOD, for which the corresponding mock control was also ≥LOD (indicating high non-specific background in a sample): In this case, samples will be adjudicated as positive for a peptide-specific response, only if the response ratio for peptide-to-mock control was ≥1.69.

### Validation of intra- and inter-assay precision

Precision was validated using PBMCs from three CMV peptide-responsive monkeys. The animals were selected to cover a range of medium to high responses to one of the cynomolgus monkey-specific CMV peptide pools and used for precision determination as follows: monkey 10 had a low response to UL83 (inter-assay mean: 74 SFU/well), monkey 16 had a medium response to UL55 (inter-assay mean: 212 SFU/well), and monkey 13 had a high response to UL83 (inter-assay mean: 405 SFU/well).

Precision was assessed by performing six assay runs, each with PBMCs from three animals and corresponding monkey CMV peptide pools. In each run, 200,000 PBMCs/well were plated and stimulated in triplicate wells. The intra-triplicate precision ranged from 2.2 to 14.3%CV (Table 2), which passed acceptance criteria (CV ≤ 30% for responses ≥ 30 SFU/well). Intra-assay precision was assessed as the average intra-triplicate %CV for individual responses from all six runs and ranged from 5.5 to 10.9%CV, which also satisfied acceptance criteria (CV ≤ 30% for responses ≥ 30 SFU/well). Inter-assay precision was assessed across triplicate mean responses from all six runs and ranged 9.9–11.3%CV, which met acceptance criteria (CV ≤ 50% for responses ≥ 30 SFU/well). Inter-analyst precision was also assessed and determined across the mean responses obtained for the same samples by two different analysts and ranged from 1.5 to 9.6%CV. These results demonstrate the consistency and precision of measured PBMC responses to stimulating peptide antigens.

**Table 2:** Validation of assay precision using UL83 and UL55 stimulation.

Monkey ID	Peptide pool	Analyst	Run	Triplicates	Intra-assay		Inter-assay		Inter-analyst	
				Mean SFU/well	%CV	Average triplicate %CV	Mean SFU/well	%CV	Mean SFU/well	%CV
10	UL83	A	5	80	12.9	10.9	74	9.9	78	6.3
			6	79	11.2					
			10	74	14.3					
		B	9	60	6.1				71	
			11	75	8.2					
			13	78	12.9					
16	UL55	A	5	208	7.8	5.5	212	11.3	214	1.5
			6	197	4.7					
			10	238	2.2					
		B	9	174	4.2				210	
			11	228	7.0					
			13	228	6.9					
13	UL83	A	5	389	8.3	5.9	405	10.9	433	9.6
			6	480	2.4					
			10	429	5.3					
		B	9	372	4.1				378	
			11	358	6.0					
			13	404	4.1					

PBMCs from three CMV-responsive monkeys (low, medium and high responders) were stimulated with monkey CMV peptide pools UL83 or UL55 in six different runs per monkey. Data were generated by two analysts, with three runs per analyst on three different days.

### Validation of assay linearity

Assay linearity describes the ability to obtain results that are directly proportional to the concentration of the measured analyte within a defined range. When applied to ELISpot assays, the number of plated, responsive PBMCs should be directly proportional to the SFU measured for IFN-γ secretion. Therefore, the PBMC plating density was varied to assess the linear range. Using PBMCs from a medium to strong responder to CMV peptides UL83 (Animal 16), ELISpot was performed in triplicate wells in three different runs at seven different cell densities (from 400,000 to 6250 cells/well in runs 1 and 3, and 400,000–8188 cells/well in run 2). The DMSO mock control was also included at a constant cell density of 200,000 cells/well (runs 1 and 3) and 262,000 cells/well (run 2). Log_10_-transformed values were plotted for cell density on the *x*-axis and mean SFU/well response under UL83 stimulation on the *y*-axis for each run. The linear range was defined as the range of cell densities through which linear regression yielded a coefficient of correlation (*r*^2^) ≥ 0.95. Cell densities with responses < LOD were excluded from linearity analysis. As shown in Table 3, the linear range was 25,000–200,000 cells/well for run 1, 8250–264,000 cells/well for run 2, and 12,500–200,000 cells/well for run 3. In summary, the validated linear range of the assay inclusive of all three runs was determined to be from 25,000 to 200,000 cells/well.

**Table 3:** Validation of assay linearity.

Monkey ID	Run	Peptide pool	Coefficient of correlation (*r*^2^)	Linear range (cells/well)
16	1	UL83	0.972	25,000–200,000
16	2	UL83	0.977	8250–264,000
16	3	UL83	0.964	12,500–200,000
	All	UL83	0.969	25,000–200,000

The linear range of the assay response was determined in three different runs with varying cell densities (cells/well) under UL83 peptide stimulation.

### Validation of assay specificity

Assay specificity was validated by stimulating CMV-responsive PBMCs from a monkey with an HIV peptide pool, to which they should remain unresponsive. ELISpot was performed in three different runs to measure the response to the HIV peptide pool tested at two concentrations: 2 μg/mL and 1 μg/mL. Stimulation with CMV peptides UL55 and UL83 was performed alongside as a peptide-responsive control, in addition to the DMSO mock control and PHA-L stimulation. To meet acceptance criteria for specificity, the PBMC response to the HIV peptide pool should remain below the LOD (15 SFU/well) or show an antigen-specific to mock response ratio of <1.69 in cases where both the peptide response and mock-control response were ≥LOD. As shown in Table 4, responses to the HIV peptide pool remained <LOD at any concentration in all three runs, similar to the DMSO mock control. However, responses to UL55, UL83, and PHA-L were consistently ≥LOD. These results demonstrate that the ELISpot assay can specifically detect cellular immune responses following stimulation with antigenic peptide pools.

**Table 4:** Validation of assay specificity.

Monkey ID	Stimulation	Run 1	Run 2	Run 3
		Mean SFU/well		
16	Mock (DMSO)	1	5	3
16	HIV (2 μg/mL)	2	3	2
16	HIV (1 μg/mL)	1	6	5
16	UL55 (1 μg/mL)	155	204	228
16	UL83 (1 μg/mL)	784	924	923
16	PHA (0.25 μg/mL)	470	551	545

PBMCs from a CMV-responsive monkey were stimulated with CMV peptide pools UL55 and UL83 or an irrelevant HIV derived peptide pool. PHA stimulation and DMSO mock control were also included.

### Assay implementation for non-clinical sample analysis

A summary of the assay validation results is provided in Table 5. The validated assay was subsequently used to monitor AAV5 capsid and transgene product (TGP)-specific T cell responses in a study where the Cynomolgus macaques were dosed with an investigational gene therapy product (two dose levels: 6E13 vg/kg and 2E14 vg/kg). A total of 72 PBMC samples were collected from 36 study animals between Day 13 and 56 after administration of the AAV5 vector and stimulated with two AAV5 peptide pools and one TGP peptide pool. Positive control stimulations included monkey CMV peptides as well as PHA, both of which were performed for each sample on all plates to verify that PBMCs in the test sample were capable of mounting an IFN-γ response. Negative controls included DMSO mock stimulation and media-only conditions.

**Table 5:** Assay validation summary.

Assay parameter	Acceptance criteria	Validation results
LOD	The LOD is the 95th percentile distribution limit of mean SFU/well responses after DMSO mock stimulation	15 SFU/well (75 SFU/million PBMC)
Confirmatory cut point	The confirmatory cut point is the 95th percentile distribution limit of peptide/mock response ratios in naïve animals.	1.69
Precision	For responses ≥ 30 SFU/well: Intra-triplicate CV ≤ 30% Intra-assay CV ≤ 30% Inter-assay CV ≤ 50%	Intra-triplicate CV: 2.2–14.3% Intra-assay CV: 5.5–10.9% Inter-assay CV: 9.9–11.3%
Positive control stimulation acceptance range	PHA responses > LOD	All PHA responses were >LOD.
Linearity	Report cell densities through which a linear regression of responses shows an *R*^2^ ≥ 0.95.	25,000–200,000 cells/well
Specificity	No positive response to irrelevant HIV control peptides.	Responses for up 2 μg/mL HIV peptides remained < LOD.

**Fig. 4:**
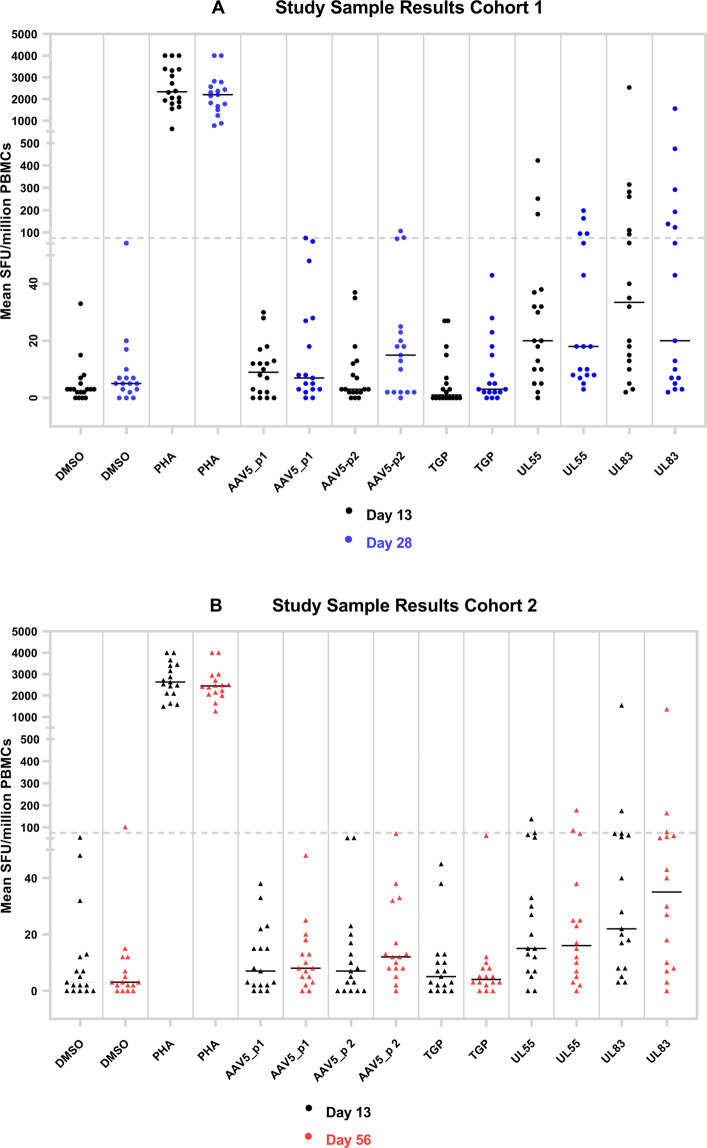
Graphical summary of cellular immune responses in study animals. PBMCs collected after AAV5 gene therapy adminstration were left unstimulated (mock) or stimulated with various peptide pools or PHA and tested in INFγ ELISpot. Respones were expressed as SFU/million PBMCs. **A** Cohort 1: PBMCs from 18 animals collected on Day 13 (black dots) and 28 (blue dots) after AAV5 gene transfer. **B** Cohort 2: PBMC samples from 18 animals collected on Day 13 (black dots) and 56 (red dots) after AAV5 gene transfer. PHA responses reported ‘as too many to count’ (TNTC) were imputed as 4000 SFU/million PBMC for graphical purposes.

A graphical summary of the results is provided in Fig. 4. The 36 study animals were allocated equally between two cohorts with different terminal time points on Day 28 (Fig. 4A) or Day 56 (Fig. 4B). Both cohorts were also assessed on Day 13. Of all study samples tested, 1 of 72 (1.4%) and 2 of 72 (2.8%) PBMC samples resulted in a positive response to AAV5 peptide pools 1 and 2, respectively. No positive responses to TGP peptide pools were identified. The positive control stimulation with PHA-L resulted in positive responses for 72 of 72 (100%) study samples, while stimulation with UL55 and UL83 peptide pools resulted in positive response for 12 of 72 (16.9%) and 18 of 72 (25.4%) study samples. Responses to monkey CMV peptide pools UL55 and UL83 were expected to occur in some but not all samples, depending on the pre-existing immune status of the study animals resulting from prior CMV exposure.

We next evaluated the magnitude and time course of the cellular immune responses against the AAV5 capsid in individual study animals. There were only two monkeys (both in cohort 1) with cellular immune responses against AAV5 that were low-positive at Day 28 (which was the terminal time point in this cohort, thus no Day 56 result was available); the response was right at the LOD in one animal and remained within 1.5 times of the LOD in the second animal (Table 6) While use of the antigen/mock response ratio was not necessary to adjudicate these positive responses, given that the mock stimulation in both animals remained below the LOD, one of the two animals also achieved a response ratio >1.69 (confirmatory cut point), thus additionally demonstrating the true-positive nature of the AAV5 response. Nonetheless, the infrequent and weak cellular immune responses against AAV5 did not correlate with any decline in transgene expression or liver transaminase (ALT) elevations (data not shown).

**Table 6:** Time course of cellular immune responses in study animals.

AAV positive responses in test samples						
		Triplicate mean SFU/million PBMCs			Cut point ratio	
Donor	Sample collection day (post dose)	Mock (DMSO)	AAV5_pool 1	AAV5_pool 2	AAV5_pool 1/Mock	AAV5_pool 2/Mock
A	D13	3	12	7	NA	NA
	D28	52	**75**	**77**	1.45	1.48
B	D13	3	12	3	NA	NA
	D28	10	48	**107**	4.83	10.67

Responses at or above the LOD (75 SFU/million PBMCs) are highlighted in *bold*. Response ratios for peptide response at or above the LOD are also shown.

*NA* not applicable.

## Discussion

Monitoring cellular immune responses that target the AAV capsid or TGP using ELISpot assays may provide insights into mechanisms that could trigger safety-related events (such as ALT elevations related to liver inflammation) or limit efficacy (decrease in expression) in non-clinical gene therapy studies. Previously, ELISpot testing has been extensively performed in exploratory research settings, where no rigorous assay performance validation may be necessary. Notwithstanding select performance characteristics, such as precision and linearity, have been evaluated previously [19, 35, 36]. Unlike immunogenicity assays to detect total or neutralizing antibodies, or ligand-binding assays to quantify drug and biomarker concentrations, there are no universally accepted procedures to validate ELISpot assays for regulated use in gene therapy studies. This creates a challenge for how to define LOD, cut points, acceptable precision, and other performance criteria. Herein we describe an approach how to validate a generic IFN-γ ELISpot assay format using monkey PBMCs and stimulation with antigenic peptide pools.

Since no formal guidelines exist for ELISpot assays, we considered some of the recommendations from FDA guidance documents and industry white papers for immunogenicity and bioanalytical assays, wherever feasible [39, 40, 43–46, 49, 50]. Generally speaking, a fit-for-purpose ELISpot assay should be sensitive, precise, reproducible between operators, specific, and semi-quantitative; hence, these parameters constituted the focus points of our validation. In the literature, a human IFN-γ ELISpot assay using 45 healthy donors utilized 10 SFU/well with 200,000 PBMCs per well as a suitable cutoff for determining a test result to be positive or negative [19]. IFN-γ ELISpot studies in monkeys have shown low background signals under pre-immune conditions (10–50 SFU/million PBMCs) and moderate precision (intra- and inter-assay CV of 21.9% and 24.7%, respectively) [51]. Even though our assay used a lower number of replicates than this previous study, we achieved even higher precision during validation, using three monkeys representing a range from low, medium to high responses to CMV (Table 3). Nonetheless, we generally consider it acceptable for ELISpots to maintain intra-assay assay CVs ≤ 30% for PBMC responses ≥ 30 SFU/well. Responses below 30 SFU/well do not yield meaningful CV values and would be interpreted as “low”, regardless of the exact numerical results (see below). In contrast, responses above 30 SFU/well may be further interpreted as “medium” or “high” and thus require precision to remain ≤30%CV. This semi-quantitative characterization of the magnitude of cellular immune response will enable a better correlation of ELISpot responses with other, orthogonal study assessments. It is therefore critical to validate ELISpot assay precision by including low, medium and high responses to antigenic peptides to cover the full range of potential test results. We propose that a low response in monkey PBMCs would be below 50 SFU/well (<250 SFU/million PBMC), a medium response would be within 50–200 SFU/well (250–1000 SFU/million PBMC), and a high response would be above 200 SFU/well (>1000 SFU/million PBMC).

A statistical approach using parametric calculations was employed for validated LOD determination. The 95th percentile distribution limit, or 1.645 times the standard deviation over the mean was chosen to set the LOD, so that the targeted false-positive rate was 5%. This conservative approach is in accordance with screening cut point establishment recommended by regulatory guidance documents for other immunogenicity monitoring assays (FDA/CBER/CDER Jan 2019) and results in a lower detection cutoff for positive responders compared to other approaches, such as defining the LOD as three times above the SFU of the unstimulated control [8, 31, 52]. Therefore, our approach allows for better detection of low positive responders, as illustrated by the non-clinical case study (Fig. 4): The two positive responders in this study would have been missed by using a higher detection cutoff. This conservative approach was also deemed more appropriate over standard practice to detect the anticipated low T cell responses in monkeys, given that cellular immune responses in previous AAV studies have been reported as minimal [4, 8]. Despite using a different statistical approach, our LOD value corresponded well with other published validated LODs [36].

To account for the increased false positive screening rate, we also established a confirmatory cut point to evaluate samples with higher background, given that some PBMC samples yielded responses > LOD even under mock stimulation. Spontaneous IFN-γ release in ELISpot assays has been described in the literature and was associated with PBMCs from individuals with elevated levels of pre-existing immune activation of both CD4+ and CD8+ T cells [53]. To discern peptide-specific responses in PBMC samples from high non-specific background, we validated a confirmatory cut point using the responses ratio between a peptide-stimulated and mock-stimulated PBMC sample. This confirmatory cut point will only be used to determine the specificity of a peptide response, if both peptide and mock stimulation resulted in responses ≥ LOD. In all other cases, a response to peptides ≥ LOD will suffice to adjudicate the samples as positive, in order to preserve the maximal sensitivity of the assay.

Based on the described validation results, the IFN-γ ELISpot assay was deemed fit-for-purpose and used in a monkey AAV5 gene therapy study. When employed for detection of gene therapy-directed cellular immune responses, the assay continued to show sensitive, precise, and specific performance. In this study, all animals developed anti-AAV5 antibodies after administration of the gene therapy vector (data not shown), thus confirming the induction of a humoral immune response against the capsid. T cell responses typically peak at 7–15 days after initial antigen stimulation, followed by memory cell formation for long-term immunity [54]. Memory T cells remain detectable in ELISpot assays, since they can be re-stimulated with peptide antigens. Surprisingly, we identified only 2 of 36 monkeys that were low positive on Day 28 post-dose for cellular immune responses against the AAV capsid. At the remaining time points (Day 13 and 56), no ELISpot responses were detected, suggesting that no significant T cell immune responses to AAV5 or TGP had been induced in most study animals. As a control, we frequently observed positive responses in PBMCs stimulated with CMV-derived UL55 or UL83 peptides, demonstrating that the assay can detect re-activated T cells against viral antigens.

Further, there was no correlation of the detected low-positive T cell responses against the AAV5 capsid with TGP expression or other laboratory tests, such as liver transaminase plasma levels. AAV-directed immunity shows a similar trend in humans, where capsid-specific cellular responses are generally less frequent or less detectable than humoral responses [55]. Based on the results herein, we conclude that cellular immune responses evoked by the AAV5 capsid may be even less frequent in monkeys than has been described for humans [1, 56, 57]. When cellular responses did occur, they did not lead to a substantial loss of liver cells that would be associated loss of efficacy or safety signals. Interestingly, recent studies indicated that selectively enriching and expanding sub-populations of PBMCs prior to ELISpot testing can offer better resolution to identify cellular immune responses to AAV (Vandamme et al., [30]; Verdera et al. [55]). These innovative approaches could be valuable and should be considered for next-generation immune-monitoring assays used in nonclinical studies.

ELISpot assays are typically performed in a 96-well or 384-well plates pre-coated with an anti IFN-γ antibody, making them well-suited for high-through-put applications. In our experience, the assay was highly reproducible across analysts, laboratory test sites, or run times for stimulation with both negative (DMSO-mock) and positive controls (PHA, UL55 or UL83 peptide). We noticed, however, that the substrate development time should be strictly kept at 3 min ± 15 s to achieve uniform spot counts across different experiments. Data acquisition and analysis can be easily standardized from plate scanning to final result calculations. One limitation of ELISpot assays is that they require three consecutive days from start to finish, but the actual hands-on time per day decreases substantially after the first day.

Using the validated monkey IFN-γ ELISpot assay described herein, we found that there may not always be clear and direct impact of cellular immune responses on AAV gene therapy outcomes. We chose to focus on IFN-γ secretion, as this cytokine is a commonly used marker, broadly associated with many cellular immune responses. Simultaneous detection of multiple cytokines including Tumor necrosis factor alpha (TNF-α) and other analytes has advantages over single cytokine measurements, but we believe that this still requires a definitive identification of suitable markers with actual clinical relevance. In particular, TNF-α may also be released in a non-antigen-specific fashion by pre-activated T cells, and even by non-T cells, such as NK or NKT cells among the PBMCs. In a recent AAV5 clinical study, it was demonstrated that TNF-α monitoring, did not offer an advantage over IFN-γ monitoring, as neither of them consistently correlated with liver enzyme elevations. (Long et al., [10], in revision, unpublished data on file at BioMarin). Therefore, further evaluation of the clinical relevance of TNF-α or other cytokines is needed. Direct measurements of cytotoxicity markers (perforin or granzyme secretion) in ELISpots may be useful to supplement immune monitoring by IFN-γ. Our preliminary experiments using human PBMCs, however, indicated that these markers are not released in sufficient quantity using the ELISpot assay format described herein (data not shown). In summary, we describe the comprehensive bioanalytical validation of a reliable method to measure IFN-γ secretion in monkey PBMCs, in order to evaluate the safety and efficacy of AAV gene therapies in nonclinical studies.
